# Effects of uremic toxins on hippocampal synaptic transmission: implication for neurodegeneration in chronic kidney disease

**DOI:** 10.1038/s41420-021-00685-9

**Published:** 2021-10-16

**Authors:** Giuseppina Natale, Valeria Calabrese, Gioia Marino, Federica Campanelli, Federica Urciuolo, Antonio de Iure, Veronica Ghiglieri, Paolo Calabresi, Maurizio Bossola, Barbara Picconi

**Affiliations:** 1 Dipartimento di Neuroscienze, Neurology Unit, Università Cattolica del Sacro Cuore, Rome, 00168 Italy; 2 Department of Medicine, University of Perugia, Perugia, 06129 Italy; 3 Laboratory of Experimental Neurophysiology, IRCCS San Raffaele Roma, Rome, 00166 Italy; 4 Fondazione Policlinico Universitario Agostino Gemelli IRCCS, Rome, 00168 Italy; 5 Hemodialysis Unit, Division of Nephrology, Università Cattolica del Sacro Cuore, Rome, 00168 Italy; 6 Telematic University San Raffaele, Rome, 00166 Italy

**Keywords:** Cellular neuroscience, Neurodegeneration

## Abstract

Patients affected by chronic kidney disease (CKD) have an increased risk of developing cognitive impairment. The cause of mental health disorders in CKD and in chronic hemodialysis patients is multifactorial, due to the interaction of classical cardiovascular disease risk factors, kidney- and dialysis-related risk factors with depression, and multiple drugs overuse. A large number of compounds, defined as uremic toxins that normally are excreted by healthy kidneys, accumulate in the circulations, in the tissues, and in the organs of CKD patients. Among the candidate uremic toxins are several guanidino compounds, such as Guanidine. Uremic toxins may also accumulate in the brain and may have detrimental effects on cerebral resident cells (neurons, astrocytes, microglia) and microcirculation. The present study aims to analyze the effect of Guanidine on hippocampal excitatory postsynaptic field potentials (fEPSPs) and in CA1 pyramidal neurons recorded intracellularly. Moreover, we compared these effects with the alterations induced in vitro by CKD patients derived serum samples. Our results show an increased, dose-dependent, synaptic activity in the CA1 area in response to both synthetic Guanidine and patient’s serum, through a mechanism involving glutamatergic transmission. In particular, the concomitant increase of both NMDA and AMPA component of the excitatory postsynaptic currents (EPSCs) suggests a presynaptic mechanism. Interestingly, in presence of the lower dose of guanidine, we measure a significant reduction of EPSCs, in fact the compound does not inhibit GABA receptors allowing their inhibitory effect of glutamate release. These findings suggest that cognitive symptoms induced by the increase of uremic compounds in the serum of CKD patients are caused, at least in part, by an increased glutamatergic transmission in the hippocampus.

## Introduction

Patients affected by chronic kidney disease (CKD) have an increased risk for cognitive impairment when compared with the general population [1]. The prevalence of cognitive impairment ranges between 10% and 40% in patients with CKD and is significantly higher in patients with end-stage renal disease (ESRD) receiving chronic hemodialysis or peritoneal dialysis [1].

Multifactorial is the cause of cognitive impairment in CKD patients and in patients on chronic hemodialysis. These factors include several risk factors related to cardiovascular disease (older age, hypertension, diabetes), kidney (anemia, uremic toxins), dialysis (hypotension, inflammation) as well as depression, insomnia, and multiple drugs use [1–3].

A systematic review on cognition in ESRD patients on chronic hemodialysis showed that memory and executive function are impaired and that the domain of orientation and attention is particularly compromised [1]. This finding, together with the observation that, after kidney transplantation, there is an improvement in several cognitive performances [4], suggests that the cognitive deficits in patients on hemodialysis may be at least partially reversible [1].

**Fig. 1:**
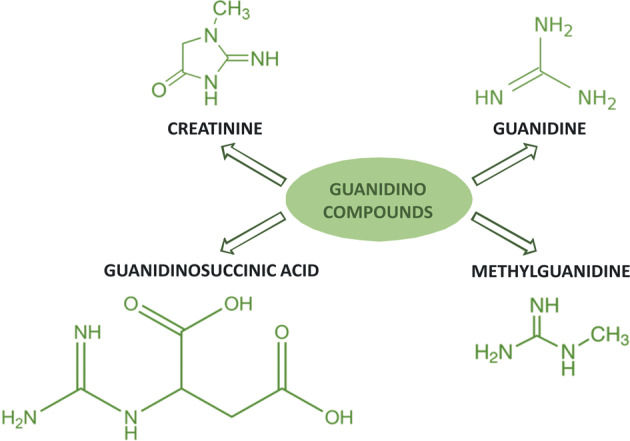
Guanidino compounds (GCs). Molecular representation of creatinine, guanidine, guanidinosuccinic acid, and methylguanidine.

A large number of compounds defined as uremic toxins, which are generally excreted by healthy kidney, accumulates in the circulations, in the tissues and in the organs of CKD and ESRD patients [5]. Uremic toxins are several guanidino compounds (GCs), such as creatinine, guanidine, guanidino succinic acid (GSA), and methyl guanidine (MG) [5] (see Fig. 1). These toxins may also accumulate in the brain [6], exerting detrimental effects on brain microcirculation and on neurons and glial cells [7]. Interestingly, pre-clinical studies using the surgical method to induce CKD, demonstrated alterations in the short-term memory and a deficit in working memory [8, 9].

Although abnormal excitatory transmission might be implicated in cognitive dysfunctions induced by GCs, experimental findings on this issue are controversial [10–12]. It has been reported that, in animal models, uremic GCs induce seizures mimicking the epileptic activity observed in the uremic brain [13]. In particular, GSA and MG, were markedly more potent convulsants than guanidine and creatinine [14]. De Deyn and Macdonald have published in the CA1 region, the increased uremic GCs levels evoked the activation of N-methyl-D-aspartate receptors (NMDARs) in conjunction with the blockade of GABA_A_ and glycine receptor-associated chloride channels [15]. In this condition, the pyramidal cells were depolarized enough to reduce the blocking action of Mg^2+^ on NMDARs, causing the influx of Ca^2+^ and the consequent increase of GSA-induced currents [15, 16]. In the present study, among the several uremic toxins accumulating in the brain of patients affected by CKD, we have analyzed the dose-dependent effect of Guanidine in bath application on field excitatory postsynaptic potentials (fEPSP) in the CA1 hippocampal area. Moreover, using patch-clamp whole-cell recordings from CA1 hippocampal pyramidal neurons, we found that Guanidine (100 μM) increases both NMDA and α-Amino-3-hydroxy-5-methyl-4-isoxazolepropionic acid (AMPA) currents suggesting a presynaptic mechanism of action for this toxin. Interestingly, the lower dose of Guanidine (1 μM) did not induce this excitatory increase of glutamatergic transmission. Finally, we found that serum of CKD patients mimics the excitatory effects induced by Guanidine, further supporting the hypothesis that increased excitatory transmission is a key factor in the development of cognitive dysfunction in CKD patients.

## Results

### Administration of synthetics Guanidine to rat hippocampal CA1 slices causes a dose-dependent increase of glutamatergic basal transmission

**Fig. 2:**
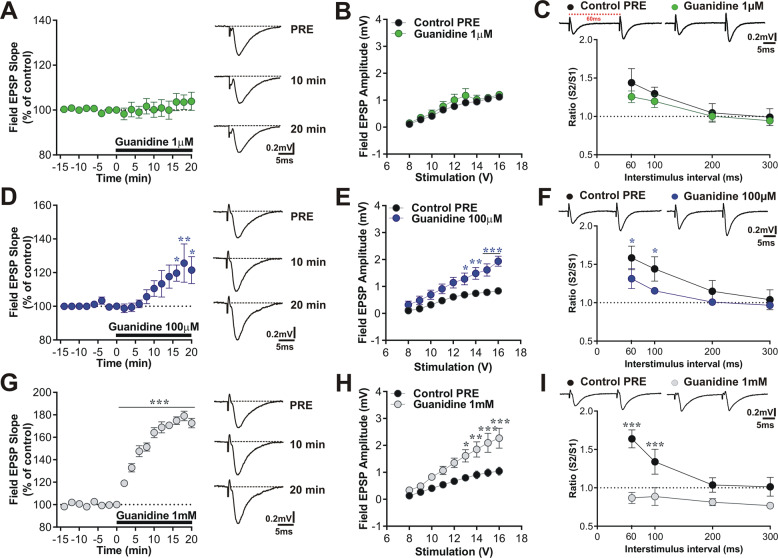
Guanidine causes a dose-dependent increase of glutamatergic basal transmission. **A**, **D**, **G** Time-course of slope fEPSP recorded from control slices in CA1 region, 10 min before and 20 min after bath application of increasing concentrations of Guanidine. Guanidine induces a dose-dependent increase in the fEPSP evoked in CA1 region (two-way ANOVA: Guanidine 100 µM, *n* = 4, F_(9,54)_ = 4.89, pre vs post 16, 18, and 20 min, Bonferroni post hoc *p < 0.05, **p < 0.01; Guanidine 1 mM, *n* = 6, F_(9,90)_ = 30.14, pre vs post 1–20 min, Bonferroni post hoc ***p < 0.001). Next to the graphs, representative traces of fEPSP PRE, 10 and 20 min after Guanidine application. Scale factor is 5 ms/0.2 mV for all traces. **B**, **E**, **H** Input/output (I/O) curves, representing variations in the fEPSP slope in response to the increasing stimulus intensity, show enhanced excitability of the CA1 fEPSPs following Guanidine application (two-way ANOVA time × group interaction: Control PRE vs Guanidine 100 µM, *n* = 7, F_(8,96)_ = 13.68, 13–16 V, Bonferroni post hoc *p < 0.05, **p < 0.01, ***p < 0.001; Control PRE vs Guanidine 1 mM, *n* = 7, F_(8,88)_ = 3.86, 13–16 V, Bonferroni post hoc *p < 0.05, **p < 0.01, ***p < 0.001). **C**, **F**, **I** Upper part; sample traces of paired fEPSPs in response to two sequential presynaptic stimuli at an interval of 60, 100, 200, and 300 ms recorded in CA1 region. Scale bars is 5 ms/0.2 mV for all traces. Graphs represent PPR before and after Guanidine 1 µM, 100 µM, and 1 mM application showing that at the 60 ms and 100 ms interval between pulses, there is a decrease in PPR parameter (two-way ANOVA time × group interaction: Control PRE vs Guanidine 100 µM, *n* = 8, F_(3,36)_ = 1.67, 60 ms and 100 ms, Bonferroni post hoc *p < 0.05; two-way ANOVA time × group interaction: Control PRE vs Guanidine 1 mM, *n* = 5, F_(3,24)_ = 20.89, 60 ms and 100 ms, Bonferroni post hoc ***p < 0.001). By contrast, the smaller dose of Guanidine (1 µM) did not alter the S2/S1 ratio (**C**).

By using extracellular recordings, we examined glutamatergic basal synaptic transmission in the CA1 hippocampal area in control slices following bath application of increasing concentrations of Guanidine (1 µM, 100 µM, and 1 mM), as previously performed by De Deyn and collaborators [15]. As shown in Fig. 2, bath application for 20 min of Guanidine at different concentrations (1 µM, 100 µM, and 1 mM) induced a dose-dependent increase in the slope of the fEPSP evoked in the CA1 region (Fig. 2A, D, G, two-way ANOVA: Guanidine 100 µM, *n* = 4, F_(9,54)_ = 4.89, pre vs post 16, 18, and 20 min, Bonferroni’s post hoc test *p < 0.05, **p < 0.01; Guanidine 1 mM, *n* = 6, F_(9,90)_ = 30.14, pre vs post 1–20 min, Bonferroni’s post hoc test ***p < 0.001). Then, the variation in the fEPSPs amplitude in response to increasing stimulus intensities was analyzed and Input/Output (I/O) responses were plotted. Analysis of the I/O relationship revealed a gradual increase on the excitability of the CA1 field potential following 100 µM and 1 mM Guanidine bath application (Fig. 2E, H, two-way ANOVA time × group interaction: Control PRE vs Guanidine 100 µM, *n* = 7, F_(8,96)_ = 13.68, 13–16 V, Bonferroni’s post hoc test *p < 0.05, **p < 0.01, ***p < 0.001; Control PRE vs Guanidine 1 mM, *n* = 7, F_(8,88)_ = 3.86, 13–16 V, Bonferroni’s post hoc test *p < 0.05, **p < 0.01, ***p < 0.001). Conversely, the lower concentration of Guanidine (1 µM) did not alter the I/O relationship (Fig. 2B).

Furthermore, we investigated changes in presynaptic transmission in the CA1 hippocampal region following Guanidine administration. At the 60 ms and 100 ms interstimulus interval, we found that the PPR in CA1 fEPSPs, recorded after 20 min-bath application of Guanidine 100 µM and 1 mM, were smaller than those recorded in basal condition (Fig. 2F, two-way ANOVA time × group interaction: Control PRE vs Guanidine 100 µM, *n* = 8, F_(3,36)_ = 1.67, 60 ms and 100 ms, Bonferroni’s post hoc test *p < 0.05; Fig. 2I, two-way ANOVA time × group interaction: Control PRE vs Guanidine 1 mM, *n* = 5, F_(3,24)_ = 20.89, 60 ms and 100 ms, Bonferroni’s post hoc test ***p < 0.001). By contrast, the smaller dose of Guanidine (1 µM) did not alter the S2/S1 ratio (Fig. 2C).

These results suggest that a presynaptic mechanism is involved in the enhanced glutamatergic basal synaptic transmission after Guanidine bath application.

### Guanidine triggers altered responses in NMDA and AMPA EPSCs in a dose-dependent manner

**Fig. 3:**
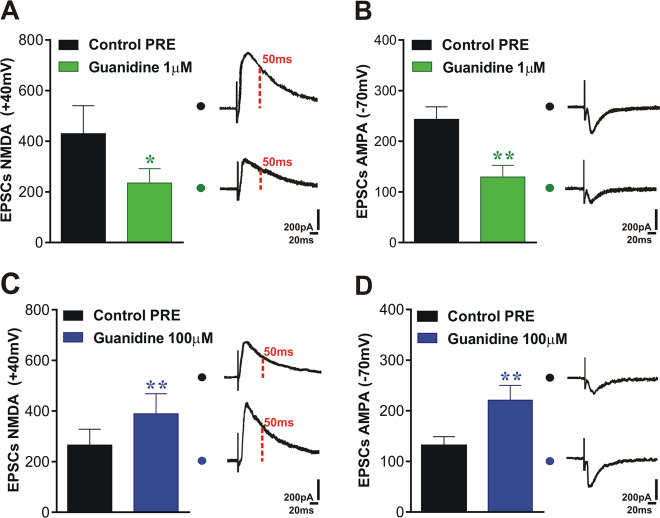
NMDA and AMPA EPSCs are altered in a dose-dependent manner after Guanidine application. **A**, **B** NMDA and AMPA EPSCs decrease after application of Guanidine 1 µM compared to the basal control (Student’s *t* test, Control PRE vs Guanidine 1 µM, *n* = 7, *t* = 3.11 df = 6, *p < 0.05 for NMDA-EPSC, *t* = 5.58 df = 6, **p < 0.01 for AMPA-EPSC). **C**, **D** Bath application of Guanidine 100 µM causes a significant increase of NMDA and AMPA EPSCs after bath application of (Student’s *t* test, Control PRE vs Guanidine 100 µM, *n* = 11, *t* = 4.00, df = 10, **p < 0.01 for both EPSCs NMDA and AMPA). Example traces from whole-cell patch-clamp experiments showing NMDAR- and AMPAR-evoked currents recorded in a CA1 neurons. Scale factor is 200 pA/20 ms for all traces.

We investigated the Guanidine (1 and 100 µM) action on glutamatergic excitatory transmission measuring NMDA and AMPA components of the EPSCs at different holding potentials (+40 mV and –70 mV). As reported in Fig. 3, we observed that the EPSCs NMDA and AMPA decreased following bath application of Guanidine 1 µM compared to control (Fig. 3A, B, Student’s *t* test, Control PRE vs Guanidine 1 µM, *n* = 7, *t* = 3.11 df = 6, *p < 0.05 for EPSCs NMDA, *t* = 5.58 df = 6, **p < 0.01 for EPSCs AMPA). Conversely, Guanidine 100 µM increased both EPSCs NMDA and AMPA (Fig. 3C, D, Student’s *t* test, Control PRE vs Guanidine 100 µM, *n* = 11, *t* = 4.00 df = 10, **p < 0.01 for both NMDA and AMPA EPSCs).

### Bath application of dialysis serum mimics the effects of Guanidine on glutamatergic transmission

**Fig. 4:**
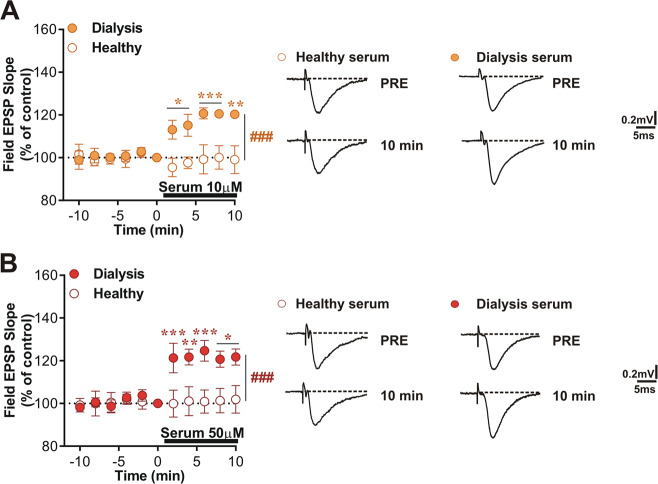
Bath application of dialysis serum mimics the effects of Guanidine on glutamatergic transmission. **A**, **B** Glutamatergic basal activity (fEPSP slope % of basal control) was increased following the in vitro application of dialysis serum (10 and 50 µM) for 10 min. **A** Two-way ANOVA: Healthy 10 µM, *n* = 4, F_(4, 24)_ = 1.05, pre vs post 1–10 min, Bonferroni’s post hoc test *p* > 0.05; Dialysis 10 µM; *n* = 5, F_(4, 32)_ = 0.46, pre vs post 2–4, 6–8, and 10 min, Bonferroni’s post hoc test *p < 0.05, **p < 0.01, and ***p < 0.001. Two-way ANOVA time × group interaction: Healthy 10 µM vs Dialysis 10 µM, F_(10,70)_ = 7.24, Bonferroni’s post hoc test ^###^*p* < 0.001. **B** Two-way ANOVA: Healthy 50 µM, *n* = 7, F_(4, 48)_ = 0.11, pre vs post 1–10 min, Bonferroni’s post hoc test *p* > 0.05; Dialysis 50 µM, *n* = 5, F_(4, 32)_ = 0.45, pre vs post 2, 4, 6, and 8–10 min, Bonferroni’s post hoc test *p < 0.05, **p < 0.01, ***p < 0.001 and two-way ANOVA time × group interaction: Healthy 50 µM vs Dialysis 50 µM, F_(10,100)_ = 9.16, Bonferroni’s post hoc test ^###^*p* < 0.001).

After the analysis of the synthetic Guanidine, which could represent just one of the several toxic GCs accumulating in the brain, we evaluated the effect of the serum of CKD patients, and healthy controls, on CA1 hippocampal excitatory transmission. We found that glutamatergic basal activity (fEPSP slope % of basal control) was increased following the in vitro application of dialysis serum at two concentrations (10 and 50 µM) with respect either to the baseline condition and healthy (10 and 50 µM) serum application (Fig. 4A, two-way ANOVA: Healthy 10 µM, *n* = 4, F_(4,24)_ = 1.05, pre vs post 1–10 min, *p* > 0.05; Dialysis 10 µM; n = 5, F(4, 32) = 0.46, pre vs post 2–4, 6–8, and 10 min, Bonferroni’s post hoc test *p < 0.05, **p < 0.01, and ***p < 0.001; two-way ANOVA time × group interaction: Healthy 10 µM vs Dialysis 10 µM, F_(10,70)_ = 7.24, Bonferroni’s post hoc test ^###^*p* < 0.001; B, two-way ANOVA: Healthy 50 µM, *n* = 7, F_(4,48)_ = 0.11, pre vs post 1–10 min, *p* > 0.05; Dialysis 50 µM, *n* = 5, F_(4,32)_ = 0.45, pre vs post 2, 4, 6, and 8–10 min, Bonferroni’s post hoc test *p < 0.05, **p < 0.01, ***p < 0.001 and two-way ANOVA time × group interaction: Healthy 50 µM vs Dialysis 50 µM, F_(10,100)_ = 9.16, Bonferroni’s post hoc test ^###^*p* < 0.001).

## Discussion

Chronic kidney disorder represents a severe risk factor for cognitive impairment development [17]. The cognitive impairment has a prevalence of 30–60% in the CKD patients with respect to age-matched controls [18]. One of the possible factors underlying cognitive decline in CKD patients is the accumulation in the blood of uremic toxins. These toxins easily cross the blood–brain barrier (BBB) and the blood-cerebrospinal fluid (CSF) barrier through specific transporters, and their high levels in the various brain regions could cause detrimental neurological effects [19]. It has been shown that, in patients with renal failure [20] or in hemodialysis patients [21], serum guanidine levels are 10 to ≥14 times higher than in control and, above all, a significant positive correlation exists between creatinine and guanidine levels in serum and cerebrospinal fluid subjects [21].

Herein, we used an electrophysiological approach to model this increase in uremic toxin content in CA1 slices of control rats. Among the several uremic toxins accumulating in the brain of patients affected by CKD, we analyzed the effect of Guanidine.

In the present study, we obtained three major findings. First, we found that Guanidine induces a dose-dependent increase of glutamatergic transmission measured by fEPSP slope. This increase of glutamatergic transmission was confirmed by the analysis of the I/O correlation, which shows the increased response of the field potential, under the same range of electrical stimulation, in the presence of Guanidine. We then moved to the analysis of pre- and post-synaptic effects of Guanidine administration. Paired-pulse ratio (PPR) changes in fEPSP responses to two subsequent stimuli is attributed to a presynaptic alteration in release probability [22, 23]. An increase in the ratio of the second pulse response (fEPSP2) to the first pulse response (fEPSP1) indicates a decrease in the release probability. The suggested reduction in the transmitter release is consistent with the observations that manipulations depressing transmitter release usually increase the magnitude of PPR [24, 25]. When the interstimulus interval is increased, the PPR decreases. In fact, the neurotransmitter release is no more influenced by the first pulse [26]. Bath application of 100 μM Guanidine, which induces a significant increase of glutamatergic transmission (as shown in Fig. 2D), affects PPR inducing an increase of fEPSP2 pulse with respect to the first one. The massive increase of glutamatergic transmission induced by the last dosage used (1 mM), is so strong that it does not anymore influence the PPR, which, in this condition, remains around 1 (fEPSP2 = fEPSP1) in all the interstimulus amplitudes.

In our whole-cell patch-clamp recordings we have not applied a GABA blocker, as is usually done, because we wanted to exclude possible epileptic effects caused by the presence of Guanidine-induced glutamatergic transmission increase and to evaluate the probability of a Guanidine influence on GABA inhibitory activity. Indeed, starting from the studies of De Deyn’s group [6, 15, 27], we hypothesized that Guanidine exerts a dose-dependent inhibitory effect on GABAergic transmission. From lower dose of Guanidine (1 μM), we obtained a decrease of NMDARs and AMPARs currents as expected in the presence of a physiological GABAergic inhibitory action. Conversely, when the recordings were made in 100 μM of Guanidine, we observed an increase of both NMDA and AMPA components of the EPSCs, further supporting the idea that this toxin might increase excitatory glutamatergic transmission at a presynaptic level, generating excitotoxicity and harming GABA inhibitory activity. This hypothesis has been already suggested by authors postulating that uremic GCs could act as competitive antagonists at the GABA_A_ receptor transmitter recognition site [10]. It should be kept in mind that the extracellular recordings, shown in Fig. 2, are much less affected by GABAergic transmission than the analysis of NMDA and AMPA currents shown in Fig. 3. Indeed, in Fig. 3A the inhibitory effect of GABAergic transmission on glutamate release can be very well appreciated.

Finally, we found that the serum derived from dialyzed patients induces a significant increase of glutamatergic transmission, thus mimicking the effect of Guanidine. This finding further supports the hypothesis of a pathological increased excitatory drive in the uremic brain. The fact that we observed this effect in the hippocampus, a brain area implicated in several memory-related processes, has an additional pathophysiological relevance.

Our results confirm the initial assumption that the serum derived from CKD patients contains guanidino compounds acting as uremic neurotoxins. These toxins might trigger excitotoxic mechanisms due to the increase of both NMDAR and AMPAR-mediated currents [27]. This combined excitotoxic mechanism, possibly associated with the inhibition of GABAergic transmission [10], could ultimately result in an abnormal increase of calcium and sodium intracellular influx, leading to neuronal death. Further studies are required to validate and clarify this hypothesis.

## Materials and methods

### Ethical approval

All procedures on animals (male Wistar rats, 2-months old) were performed in strict accordance with a protocol approved by the Animal Care and Use Committee at the Italian Ministry of Health and European Communities Council Directive of September 2010 (2010/63/E), and every effort was made to minimize animal suffering and reduce the number of animals used for the experiments (*n* = 35 rats).

### Electrophysiology

Hippocampal slices were prepared as previously described [28]. Briefly, hippocampal slices (thickness, 280–400 μm) were cut from male Wistar rats (Charles River Laboratories, Inc., Wilmington, MA, USA) using a vibratome. A single hippocampal slice was then transferred into the recording chamber and submerged with Krebs’ solution at a constant rate of 2.5 mL/min at a temperature of 30 °C, bubbled with a 95%O_2_–5%CO_2_ gas mixture. The composition of the solution was (in mM) 126 NaCl, 2.5 KCl, 1.2 MgCl_2_, 1.2 NaH_2_PO_4_, 2.4 CaCl_2_, 10 Glucose, and 25 NaHCO_3_.

#### Extracellular recordings

Recording electrodes were made of borosilicate glass capillaries (Harvard Apparatus, Holliston, Massachusetts) and filled with 2M NaCl (resistance, 10–15 MΩ). Under visual control, a bipolar tungsten-stimulating electrode (World Precision Instruments, Friedberg, Germany) was positioned into the Schaffer collateral fibers and extracellular field excitatory postsynaptic potentials (fEPSPs) were recorded with a recording electrode into the CA1 region. Testing stimuli of 0.1 Hz, 10 μs duration, and 20–30 V amplitude evoked fEPSPs that were 50–70% of maximum slope. An Axoclamp 2B amplifier (Molecular Devices, USA) was used for extracellular recordings. Input/Output (I/O) relationships were measured at the start and after drug application of each experiment by applying a series of stimuli of increasing intensity to the Schaffer collaterals. Paired-pulse ratio (PPR) indexes were calculated as mean (±SEM) as the ratio of the slope of fEPSP2nd/fEPSP1st) at various interstimulus intervals (60, 100, 200, and 300 ms).

#### Whole‐cell patch‐clamp recording

For patch-clamp recordings, neurons were visualized using infrared differential interference contrast microscopy in the CA1 region (Eclipse FN1, Nikon). Whole-cell recordings were performed with borosilicate glass pipettes (resistance, 6–9 MΩ) (Harvard Apparatus, Holliston, Massachusetts). In the voltage-clamp recordings of AMPA and NMDA receptor-mediated currents has been used pipettes filled with an internal solution containing (in mM): 120 CsMeSO_3_, 10 CsCl, 8 NaCl, 2 MgCl_2_, 10 HEPES, 0.2 EGTA, 10 TEA, 5 QX314, 0.3 NaGTP, and 2 Mg-ATP. Under visual control, a stimulating electrode was inserted into the Schaffer collateral fibers, and a recording electrode was inserted into the pyramidal CA1 region of the hippocampal slice [28].

Signals were amplified with a Multiclamp 700B amplifier, recorded, and stored on PC using pClamp 10.4 (Molecular Devices, USA). Whole-cell access resistance was 15–30 MΩ. Input resistances and injected currents were monitored throughout the experiments. Variations of these parameters >20% lead to the rejection of the experiment. For the NMDA and AMPA current experiments, neurons of the CA1 region were voltage-clamped at −70 and +40 mV to record, respectively, AMPA-mediated and NMDA-mediated EPSCs [29]. All the experiments have been done in the absence of picrotoxin, a GABA receptor inhibitor. The NMDA component of the EPSC was individuated by using the kinetic method, considering the peak amplitude at 50 ms after the beginning of the event.

### Chemicals

Synthetic uremic Guanidine compound, Guanidine hydrochloride, was from Sigma-Aldrich (USA).

The serum of the healthy subjects (*n* = 5) and dialysis patients (*n* = 10) was provided by the Hemodialysis Unit, Division of Nephrology, Università Cattolica del Sacro Cuore, Rome (Dr. Maurizio Bossola). Table 1 reported the concentration of routinely analyzed clinical parameters in healthy and dialysis serum.

**Table 1:** Demographic and clinical features of healthy subjects and dialysis patients (mean ± SD).

	Healthy subjects (*n* = 5)	Dialysis patients (*n* = 10)
Age, years	45.75 ± 6.41	55.8 ± 24.34
Sex, *n* (male:female)	1:3	7:3
Creatinine, mg/dl	0.55 ± 0.12	10.83 ± 2.93***
Urea, mg/dl	14.00 ± 2.12	80.90 ± 15.55***
Uric acid, mg/dl	4.40 ± 0.47	6.31 ± 1.72*
Phosphorus, mg/dl	3.18 ± 0.25	5.60 ± 2.02*

Differences between groups are not statistically significant except for: *p < 0.5; ***p < 0.001.

Data we reported as mean ± SD. Student’s *t* test for unpaired data was used to compare the demographic and clinical features of the experimental groups. A *p* value of <0.5 was considered statistically significant.

Guanidine and serum were applied by dissolving them to the desired final concentration in oxygenated Krebs’ solution and were bath applied by switching the standard solution.

### Statistical analysis

Animal sample size has been calculated with G*Power software (5% type I error, 80% power). The study was not classified as randomized and blinded because it does not expect random assignment of animals to treatment groups and the investigator knows the experimental procedure of each group and assesses the electrophysiological outcome. Data analysis of electrophysiological experiments was performed offline using Clampfit10.4 (Molecular Devices, USA) and GraphPad Prism 6.0 (GraphPad Software). We applied the assumption of a normal distribution with a similar variation within each group of data statistically compared.

Values in the text and figures are mean ± standard error of the mean (SEM), *n* representing the number of recorded neurons. Paired Student’s *t*-test was used for the electrophysiological analysis of the effect of synthetic uremic Guanidine in vitro application two‐way ANOVA with multiple comparisons was utilized for statistical analysis between different experimental groups over time or at a specific time point, respectively. When time × group interaction was significant, group means for each time point were compared using Bonferroni’s post hoc test. The significance level was established at *p* < 0.05.

## Supplementary information

Author contribution form

## Data Availability

The data presented in this study are available on request from the corresponding author.
