# Supplementary material for: Effects of uremic toxins on hippocampal synaptic transmission: implication for neurodegeneration in chronic kidney disease
Source: Cell Death Discov. 2021 Oct 16;7:295. doi: 10.1038/s41420-021-00685-9 (PMC8520534; doi:10.1038/s41420-021-00685-9)
Supplement: Supplementary file 1 — Author contribution form [file 41420_2021_685_MOESM1_ESM.pdf]

DECLARATION OF CONTRIBUTIONS TO ARTICLE

ADMC

Manuscript Number: CDDISCOVERY-21-1535R

Journal Name: *Cell Death Discovery* (the 'Journal')

Proposed Title of the Contribution:

Effects of uremic toxins on hippocampal synaptic transmission: implication for neurodegeneration in chronic kidney disease (the 'Contribution)

Author(s):

Giuseppina Natale#, Valeria Calabrese#, Gioia Marino, Federica Campanelli, Federica Urciuolo, Antonio de Iure, Veronica Ghiglieri, Paolo Calabresi, Maurizio Bossola*, Barbara Picconi,* (the 'Authors')

For all *CDDis*covery articles, each person named as an author in the published version must be able to show he or she has contributed substantially to the article.

Authorship credit should be based on 1) substantial contributions to conception and design, acquisition of data, or analysis and interpretation of data; 2) drafting the article or revising it critically for important intellectual content; and 3) final approval of the version to be published. Authors should meet conditions 1, 2 and 3.

Any person who cannot be shown to have made a substantial contribution to the article cannot be listed as an author in the final version. The name of any person who is deemed to have made a minor contribution can, however, appear in the Acknowledgments section of the article.

Please complete the table below to indicate the contributions of all named authors to the manuscript.

Author Full Name:	Specification of Contribution to the Manuscript:
Giuseppina Natale	Wrote the original draft of the main text, performed the experiments and the figures
Valeria Calabrese	Wrote the original draft of the main text, performed the experiments and data analysis
Gioia Marino	Performed experiments and data analysis
Federica Campanelli	Performed experiments and data analysis
Federica Urciuolo	Performed the urate serum part of the study
Antonio de Iure	Contributed to perform the electrophysiological analysis
Veronica Ghiglieri	Conceptualized the study
Paolo Calabresi	Edited and revised the final version of the manuscript
Maurizio Bossola	Conceptualized the study, wrote the first version of the manuscript, supervised the patient's urate serum analysis
Barbara Picconi	Conceptualized the study, supervised and coordinate the electrophysiological collaborators, wrote the manuscript

Please complete the table below to indicate the contributions of all named authors to the figures.

Figure 1:

Giuseppina Natale

Figure 2:

Giuseppina Natale and Valeria Calabrese

Figure 3:

Federica Campanelli, Gioia Marino and Antonio de Iure

Figure 4:

Giuseppina Natale and Valeria Calabrese

Figure 5:

Figure 6:

Signed for and on behalf of the Author(s):	Print Name:	Date:
Barbara Picconi	Barbara Picconi	July 29 2021
